# Facile Fabrication of Porous Conductive Thermoplastic Polyurethane Nanocomposite Films *via* Solution Casting

**DOI:** 10.1038/s41598-017-17647-w

**Published:** 2017-12-12

**Authors:** Tongfei Wu, Biqiong Chen

**Affiliations:** 1 Department of Materials Science and Engineering, University of Sheffield, Mappin Street, Sheffield, S1 3JD United Kingdom; 2 School of Mechanical and Aerospace Engineering, Queen’s University Belfast, Stranmillis Road, Belfast, BT9 5AH United Kingdom

## Abstract

Porous conductive polymers are one of important materials, featuring lightweight, large specific surface area and high porosity. Non-solvent induced phase separation is widely employed to prepare porous polymer sheet materials. Through utilizing water vapor in ambient environment as the non-solvent, a facile approach was developed to produce porous conductive polymer nanocomposites using the conventional solution-casting method. Without using any non-solvent liquids, porous carbon nanofiber/thermoplastic polyurethane (CNF/TPU) nanocomposites were prepared directly by solution casting of their dimethylformamide (DMF) solutions under ambient conditions. The strength of the CNF framework played a key role in preventing the collapse of pores during DMF evaporation. The dependence of porous structures on CNF loading was studied by scanning electron microscopy and porosity measurement. The influence of CNF loading on the mechanical properties, electrical conductivity and piezoresistive behavior was explored.

## Introduction

Thermoplastic polyurethane (TPU) is a kind of multi-block copolymers, typically synthesized from a di-isocyanate and a long-chain diol with a small molecule diol as the chain extender^1^. Through end-use-oriented choice of the di-isocyanates and diols, a huge number of TPUs with various physical properties have been developed^2^. TPU elastomers feature soft segments with glass transition temperature (*T*_*g*_) lower than room temperature (typically, −30 ~ 20 °C)^3^. The hard segment comprises rigid backbone moieties, rich of potential hydrogen-bonding sites (*i.e.* urethane groups), forming the hard domain through microphase separation, and serving as the physical cross-linkage in TPU elastomers^4^. Owing to their mechanical robustness, high resilience, small compression set, and excellent resistance to impacts, abrasions, tears, and weather, TPU elastomers have been extensively used over the fields of coatings, footwear, automotives and biomedical industry^5^.

Recently, electrically conductive TPU composites have arisen comprehensive interest in electrical areas like antistatic, electromagnetic shielding and sensing materials^6^. To prepare conductive TPU composites, embedding conductive fillers (*e*.*g*. conductive carbon black, nanotubes, nanofibers, graphene, silver nanowires, metal microparticles) within TPU elastomers is one attractive method, in terms of the advantages of manufacture simplicity, cost-effectiveness, and tuning of conductivity^7^. However, it is difficult to develop highly conductive TPU composites by increasing the loading of conductive fillers and simultaneously conserve the outstanding resilience originating from TPU elastomers^8^. This drawback will eventually worsen the stretchability and durability of the material. Porous structures (*e*.*g*. foams, sponges, aerogels), which feature lightweight, large specific surface area and high porosity, have been explored as a promising measure to tackle this issue. The application of porous materials have been widely spread over biological scaffolds^9^, catalyst carriers^10^, membrane filters^11^, thermal insulators^12^, super chemical adsorbents^13^, and energy absorbers^14^. Meanwhile, porous conductive polymer composites have demonstrated great potential in novel electronics, including electromagnetic interference shielding^15^, triboelectric generators^16^ and piezoresistive sensors^17^. The introduction of appropriate porous structures not only helps to effectively reduce the density and cost, but also enables the materials with improved flexibility, stretchability, and strain at break.

There are many approaches to introduce porous structures to TPU elastomers, such as *in situ* polymerization/batch foaming^18^, extrusion foaming^19^, template leaching^20^, phase inversion^21^, solution blowing^22^, thermally induced phase separation^23^, selective laser sintering^24^, and electrospinning^25–27^. Most of these methods can be readily employed to develop porous conductive TPU composites. For instance, Li *et al*. printed macroporous CNT/TPU composite materials from CNT-coated TPU particles using selective laser sintering^28^. The resulting materials exhibited high conductivity, good flexibility and great durability. The electrical conductivity showed a low percolation threshold of 0.2 wt.% and 0.1 S/m was achieved at 1 wt.% CNT loading. Lately, Hou *et al*. reported a set of porous graphene/TPU composites prepared by the infiltration of TPU into graphene scaffolds^29^. A maximum graphene loading of 10 wt.% was achieved, and the porous graphene/TPU composites showed high compression modulus and low thermal conductivity. Porous conductive CNT/TPU composites were also developed by using carbon dioxide as foaming agent^30^. It was found the percolation threshold of porous CNT/TPU nanocomposites was higher than their solid counterparts, attributed to the volume expanding. Freeze drying method is a combination of thermally induced phase separation and template-leaching-like techniques. Porous conductive CNT/TPU and graphene/TPU composites prepared by freeze drying exhibited high porosity (up to 90%) and well-defined piezoresistive behavior^17,31,32^. Phase inversion, involving non-solvent induced phase separation, is considered as a facile and low-energy-consuming approach to prepare membranes with well-defined cell structures^33^. It was found that when exposed to water vapor, TPU dissolved in dimethylformamide (DMF) and N-methyl-2-pyrrolidone (NMP) would be precipitated. If the concentration was high enough, the TPU solution would transform into an organogel. But, the porous structure in the TPU organogel could not be held during the evaporation of solvent because of the evaporation-induced shrinkage, as illustrated in a following section. To avoid the collapse of the porous structure, Chen *et al*. immersed the organogel into non-solvent water to solidify the framework and the resultant porous graphene/TPU composites exhibited lower modulus, larger elongation at break, and lower hysteresis^7^.

In this study, to avoid the collapse of the porous structure in TPU organogel, we utilized carbon nanofibers (CNFs) to cement the porous structure and developed a facile approach to produce porous conductive TPU nanocomposite films using the conventional solution-casting method. Without using any non-solvent liquids, porous CNF/TPU nanocomposites were prepared directly by solution casting of their DMF solutions in the ambient environment. The porous structures and their dependence on CNF loadings were studied by using scanning electron microscopy (SEM) and porosity measurement. The influences of CNF loading on the mechanical properties, electrical conductivity and piezoresistive behavior of the porous nanocomposites were investigated. To the best of our knowledge, few studies have declared the preparation of microcellular polymer nanocomposites directly by solution casting. This study provides a novel and facile approach to produce lightweight conductive polymer films.

## Results and Discussion

### Preparation of porous CNF/TPU nanocomposites

**Figure 1:**
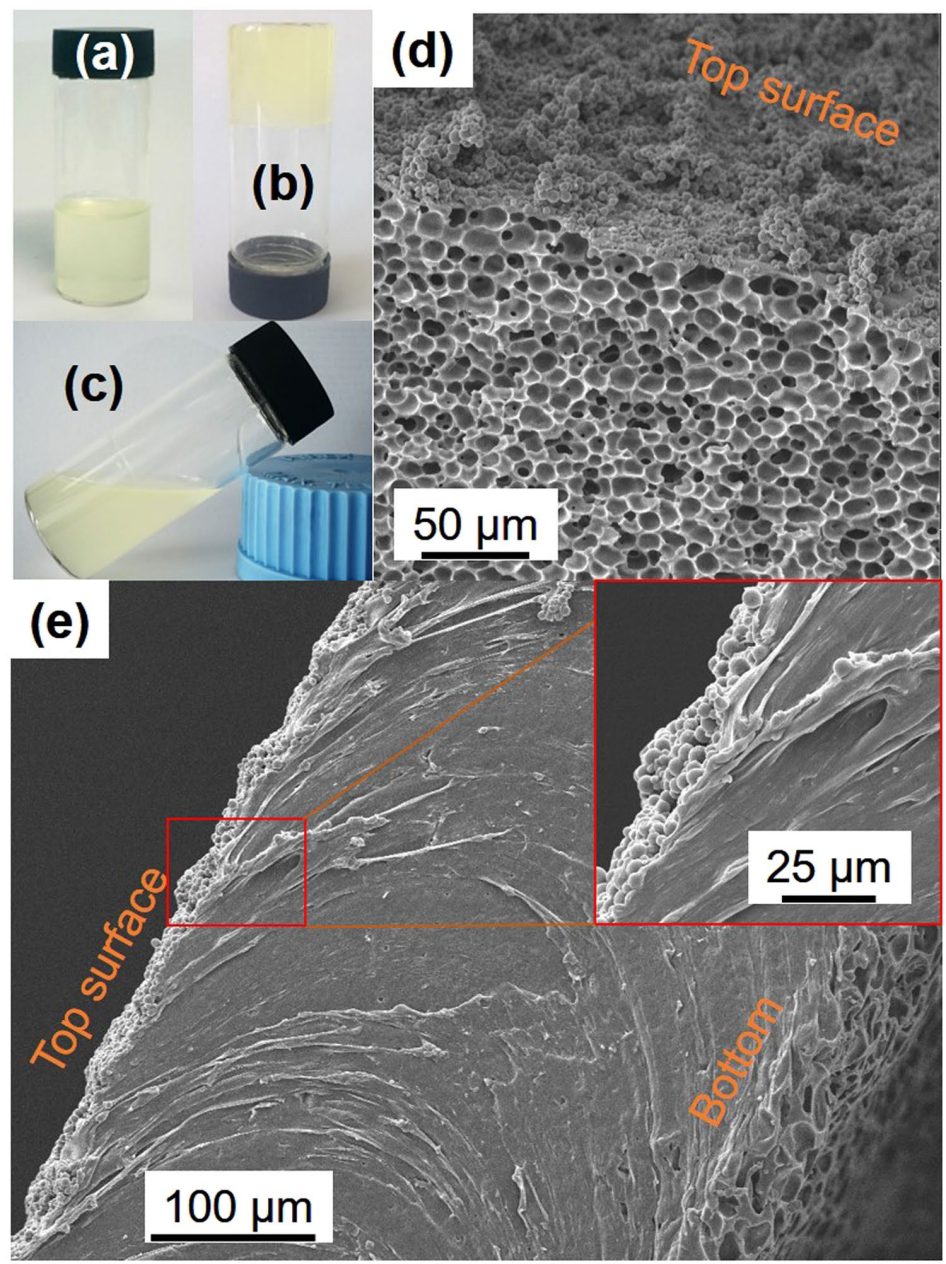
Digital photographs of a TPU DMF solution (0.08 g/mL) (**a**) and TPU DMF organogel at room temperature (**b**) and at 50 °C (**c**). Cross-section SEM images of TPU samples prepared from TPU organogel by solvent exchanging with water (**d**) and DMF evaporation (**e**).

As discussed above, TPU DMF solution is not stable when exposed to the humid air at room temperature, which is known as water-vapor induced phase separation^7^. Figure 1a illustrates a TPU DMF solution (0.08 g/mL). Once removing the cap, the gelation occurred under the ambient conditions, starting at the liquid surface and extending into the depths beneath. In this case, the gelation fully completed in 12 h and the resultant TPU DMF organogel is shown in Fig. 1b. The TPU DMF organogel was found thermally responsive such that the sample enclosed in a vial could regain the capability to flow at a temperature higher than 50 °C (as shown in Fig. 1c) and recovered after cooled down to room temperature. To obtain TPU sheets, we removed DMF from the TPU DMF organogel using two methods. One was solvent exchanging with water (immersing the organogel in water followed by drying to remove water); the other was allowing the evaporation of DMF in a fume cardboard at room temperature. The cross-section SEM images of the resultant two TPU sheets are shown in Fig. 1d and e. It can be seen that, without the solidification using water, the porous structure in the TPU DMF organogel was not able to retain after the evaporation of DMF (Fig. 1e). It was because that the TPU-phase framework in the organogel was not strong enough to withstand the shrinkage caused DMF evaporation. By contrast, owing to the restriction from the container, the uneven bottom surface of the DMF-evaporation sample still preserved the traces of the porous structure in the TPU DMF organogel. It was also noted that the top surfaces of both samples were fully covered by numerous uniform, connected TPU micropartiles (size ~5 µm), suggesting the growth of TPU DMF organogel by coalescence processes^34^. The morphologies derived from TPU aggregation at the liquid surface and in the bulk liquid phase were different, which might be related to the diffusion of water vapor and the convective flow induced by DMF evaporation.

**Figure 2:**
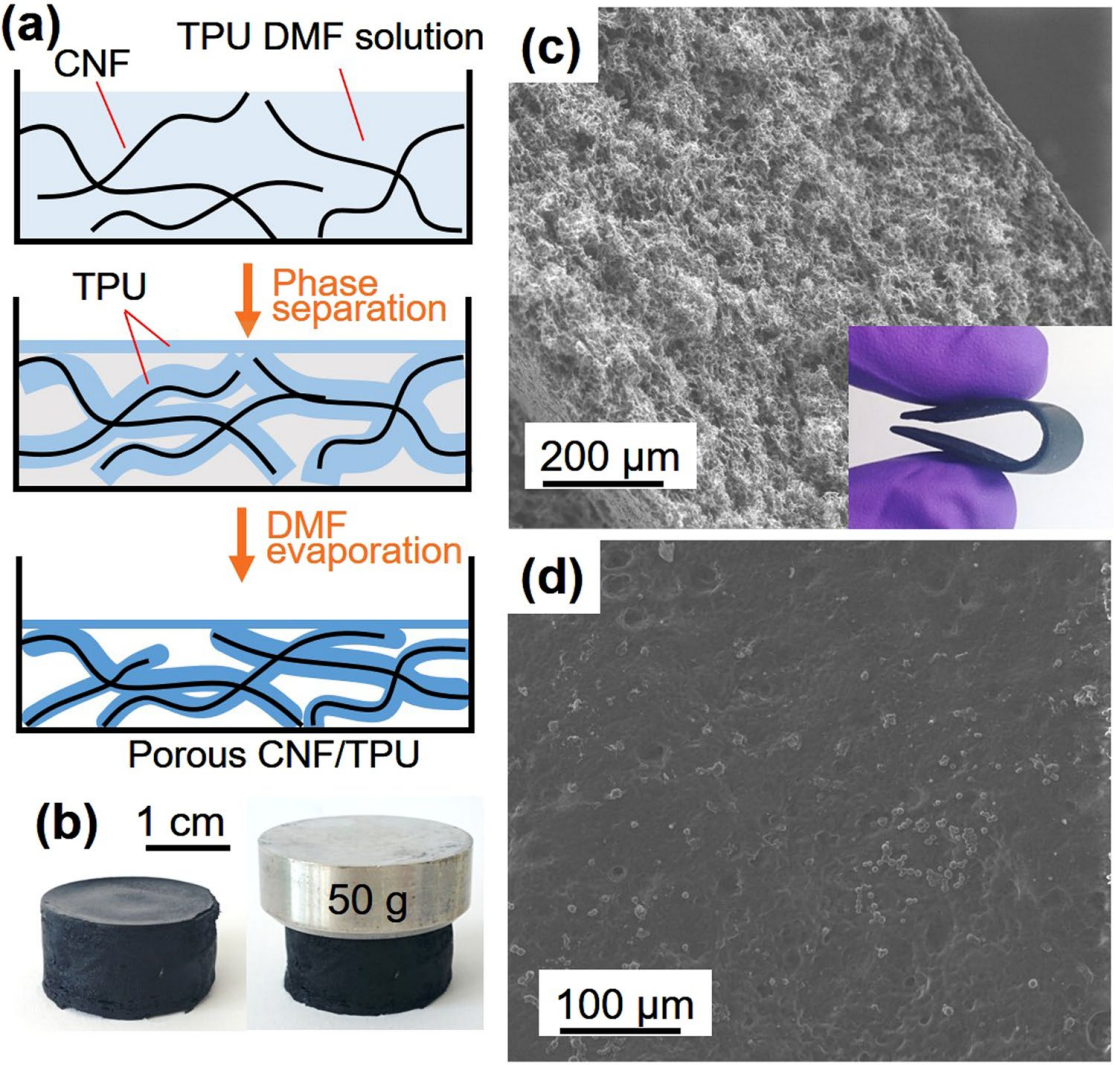
(**a**) Schematic illustration of the pathway to prepare porous CNF/TPU nanocomposites by solution casting. (**b**) Photographs of a self-standing CNF/TPU DMF organogel (0.3 g/0.7 g/15 mL). SEM images of TPU30: the cross section (**c**) and top surface (**d**). The inset of c is a photograph of TPU30.

**Figure 3:**
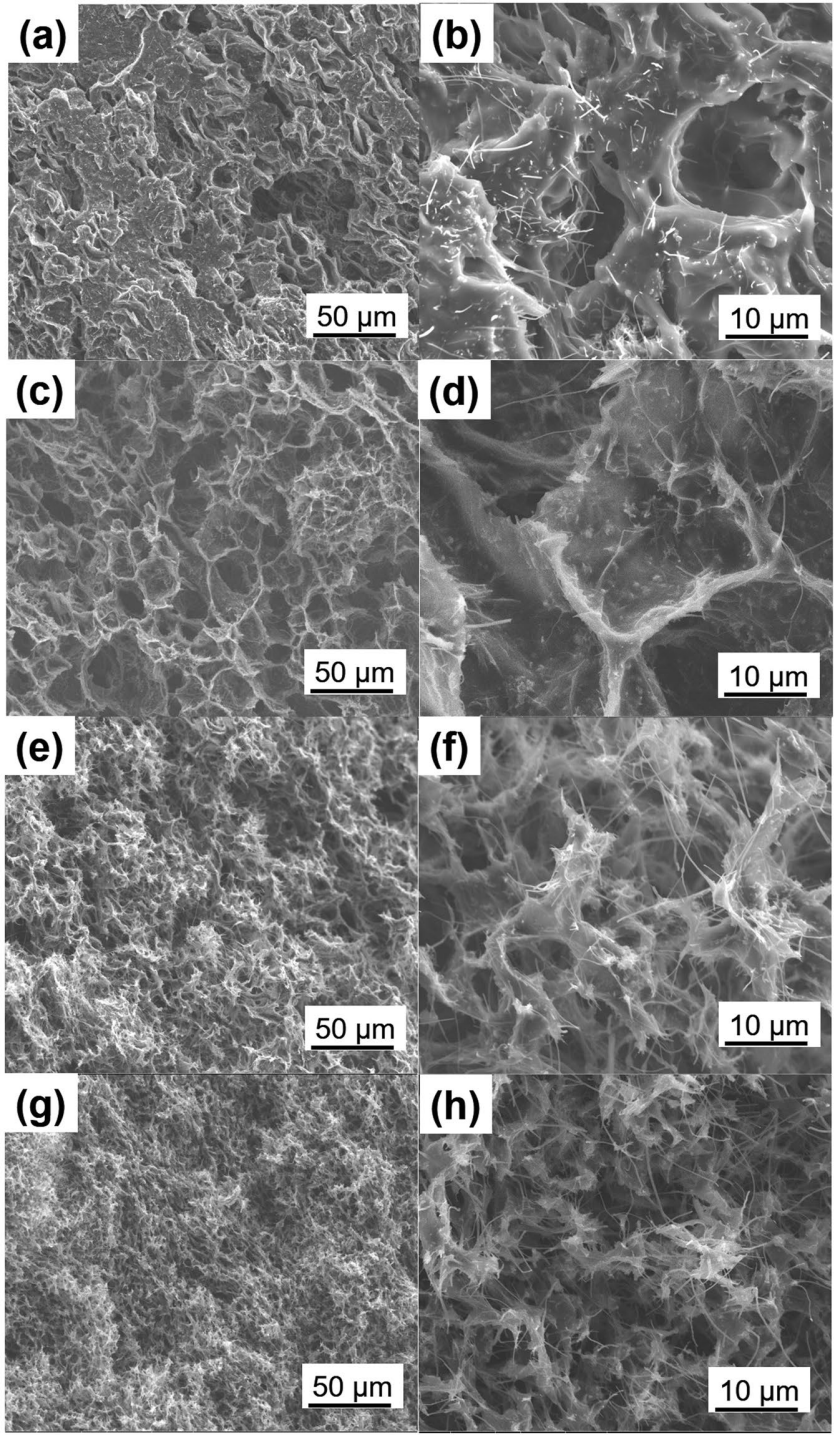
Cross-section SEM images of CNF/TPU nanocomposites: TPU10 (**a**,**b**), TPU20 (**c**,**d**), TPU30 (**e**,**f**), and TPU40 (**g**,**h**).

**Figure 4:**
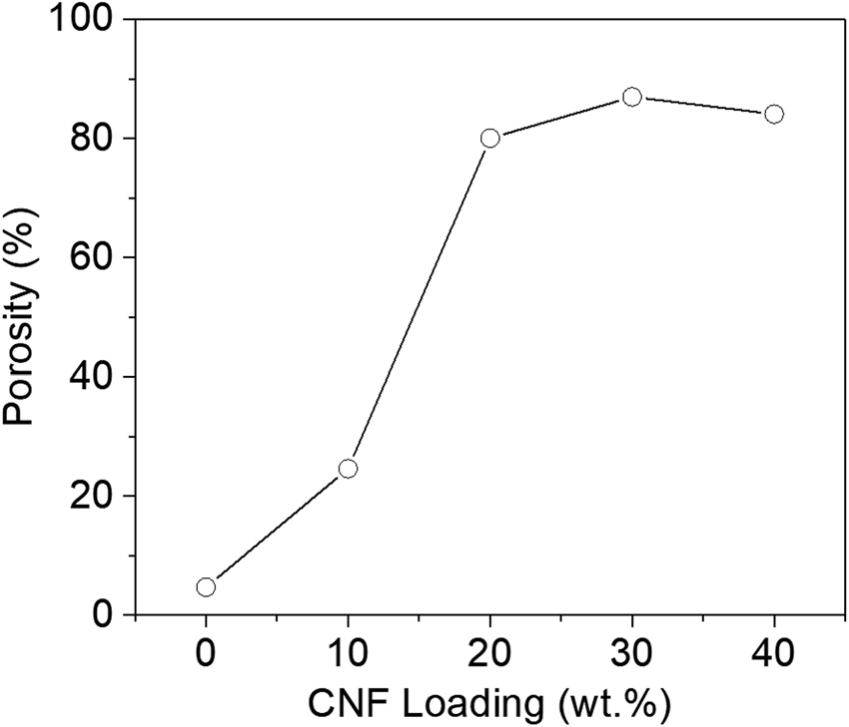
Porosity of TPU and CNF/TPU nanocomposite samples.

Rigid conductive fillers were proposed to cement the TPU-phase framework in the organogel so that porous structures could be achieved using the conventional solution-casting method. Figure 2a illustrates the pathway to utilize CNFs to reinforce the organogel and prepare porous CNF/TPU nanocomposites directly by solution casting. The obtained CNF/TPU DMF organogel was quite strong as shown in Fig. 2b. After removing DMF through evaporation, the resultant CNF/TPU nanocomposite sheets were porous and flexible, as shown in Fig. 2c. It can be seen from Fig. 2d that the top surface of CNF/TPU nanocomposites was smoother than that of the neat TPU sample as shown in Fig. 1e. This indicates the presence of CNFs significantly suppressed the formation of TPU microparticles at the liquid surface because they spatially blocked the coalescence of small TPU nucleus particles^34^. A set of CNF/TPU nanocomposites with various CNF loadings (*i*.*e*. 10, 20, 30 and 40 wt.%) were prepared using this method. Their porous structures were investigated by SEM, as shown in Fig. 3. The straight wires in SEM images are individual CNFs. The CNF/TPU nanocomposite with 10 wt.% CNF (TPU10) exhibited underdeveloped porous structures (Fig. 3a,b), indicating that the CNF-reinforced TPU framework at 10 wt.% CNF loading was not able to fully withstand the shrinkage caused DMF evaporation. With further increasing CNF loading, the porous structures in CNF/TPU nanocomposites became well-developed. From Fig. 3(c–h), TPU20, TPU30 and TPU40 showed a well-defined open-cell structure and the pore size decreased with increasing CNF loading. The porosity (void fraction) of CNF/TPU nanocomposites is shown in Fig. 4. It can be seen the porosity significantly increased when increasing CNF loading to 20 wt.% and reached a plateau at approximately 80%.

FT-IR spectra were analyzed in order to elucidate the chemical structure of TPU, CNFs and CNF/TPU nanocomposites, as shown in Fig. S1 and Table S1 in SI. TPU exhibits the characteristic IR absorptions of polyether-diol based polyurethanes: 3330 and 3340 cm^−1^ (N-H stretching in carbamate), 2970 and 2950 cm^−1^ (C-H stretching), 1730 cm^−1^ (C=O stretching in carbamate), 1596 cm^−1^ (C=C stretching in benzene ring of the rigid isocyanate segment), 1533 cm^−1^ (amide II in carbamate), 1464 and1413 cm^−1^ (CH_2_ vibrations in polyether diol), 1360 cm^−1^ (CH_3_ wagging), 1309 cm^−1^ (aromatic C-N vibration in carbamate), 1254 cm^−1^ (amide III in carbamate), 1221 and 1160 cm^−1^ (C-O-C stretching in polyether diol)^35,36^. It was noted that TPU showed only one well-defined peak at 1730 cm^−1^, corresponding to free C=O^37^, indicating most of carbonyl groups in TPU were not involved in hydrogen bonding. For TPU cast from its THF solution, 56% of carbonyl groups were involved in hydrogen bonding^37^. The difference in the hydrogen-bonding state of carbonyl groups might be related to the solvent used for solution casting. CNFs show no obvious IR absorption peaks and the percent transmittance monotonically increases with the wavenumber decreasing, due to their highly graphitic structure, which is in great agreement with previous report^38^. The percent transmittance was significantly reduced with the introduction of CNFs and decreased with increasing CNF loading. Resonant Mie scattering (RMieS) was observed^39–41^, indicated by the pronounced oscillation of the baseline around 1740 and 2970 cm^−1^. This suggests the thickness of TPU phase coated on CNFs decreased with increasing CNF loading which is consistent with the SEM observations.

### Mechanical properties of porous CNF/TPU nanocomposites

**Figure 5:**
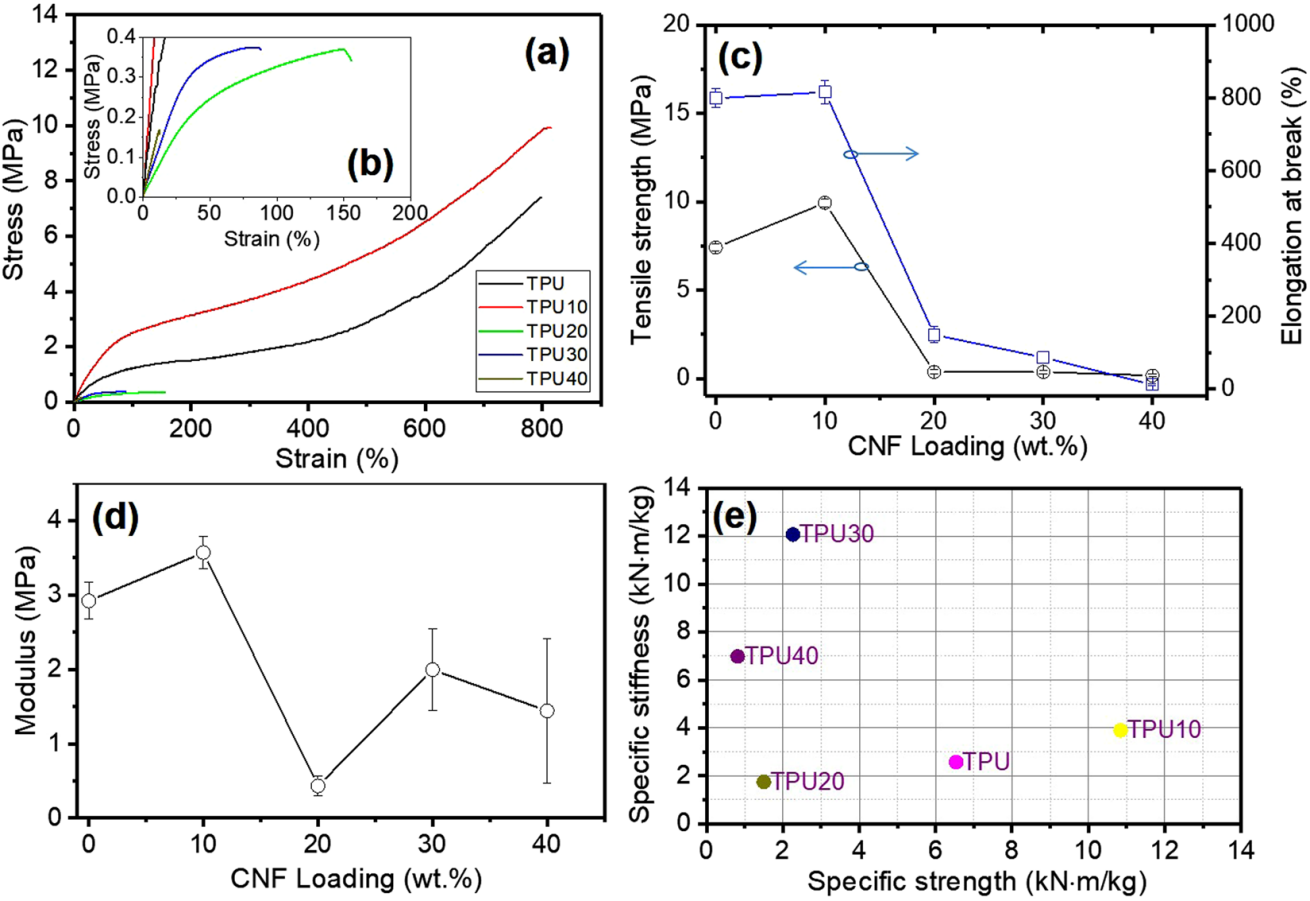
Tensile properties of porous CNF/TPU nanocomposites: representative tensile stress-strain curves (**a**,**b**), ultimate tensile strength and elongation at break (**c**) and Young’s modulus (**d**). (**e**) Specific stiffness vs. specific strength for porous CNF/TPU nanocomposites.

Mechanical properties of porous CNF/TPU nanocomposites were investigated by tensile testing. Figure 5a and b show the tensile stress-strain curves. The averaged results are summarized in Fig. 5c and d, with the error bars referring to standard deviations. TPU is not an ideal linear viscoelastic material, where the modulus changes with the strain^42^. Ultimate tensile strength, elongation at break, and Young’s modulus (at initial linear stage) were 7.41 MPa, 798%, and 2.92 MPa for neat TPU, respectively. In comparison to neat TPU, tensile strength and Young’s modulus of TPU10 (with a porosity of 24.3%) were raised by 34% and 22%, respectively, due to the reinforcement effect of CNFs. For highly porous samples, namely TPU20 (80.1% porosity), TPU30 (87.3% porosity) and PU40 (84.5% porosity), their tensile strength and Young’s modulus were remarkably lower than that of neat TPU and TPU10 owing to high porosity. It was noticed that there was no clear trend in the Young’s modulus. This was caused by the fact that a higher CNF loading is expected to lead to a higher Young’s modulus in its non-porous TPU nanocomposites; yet the Young’s modulus of a porous nanocomposite is also inversely affected by the porosity which is again dependent on the CNF loading. The values of elongation at break were 810%, 149%, 87% and 12% for TPU10, TPU20, TPU30 and TPU40, respectively. The dramatic decline in elongation at break from TPU10 to TPU20 can be explained by the fact that the latter had a much higher porosity which reduced the uniformity of the material, resulting in more defects and other stress concentrations. Although these values were lower than that of neat non-porous TPU, the mechanical performance of TPU20, TPU30 and TPU40 was commendable considering such high porosities. Specific stiffness and specific strength of samples are shown in Fig. 5e. It can be seen that TPU30 presented the best stiffness-to-weight ratio while TPU10 showed the best strength-to-weight ratio.

### Electrical properties of porous CNF/TPU nanocomposites

**Figure 6:**
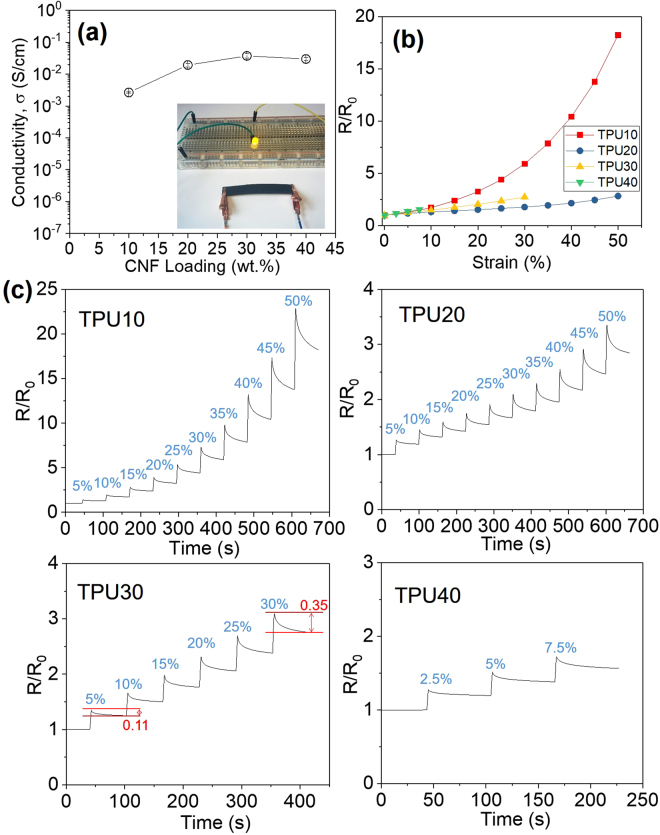
(**a**) Electrical conductivity of CNF/TPU nanocomposites versus the CNF loading. (Inset) Demonstration of the electrically conductive property of TPU40 in an LED circuit. (**b**) The normalized resistance (*R/R*_0_) of CNF/TPU nanocomposites as a function of strain. (**c**) *R/R*_0_ of CNF/TPU nanocomposites as a function of time at different strains as represented by percentages in blue.

The CNF network provides a continuous conductive network in the insulating TPU matrix. The experimentally determined electrical conductivity (σ) increases with the CNF loading, from 2.66 × 10^−3^ S/cm with 10 wt.% CNFs to 2.98 × 10^−2^ S/cm with 40 wt% CNFs (Fig. 6a). It was found that the resistance of CNF/TPU nanocomposites changed under stretching (Fig. 6b). At 50% stain, the resistance was raised by 17.2 and 1.7 times for TPU10 and TPU20, respectively. The increment at 30% stain was 5.0, 0.8 and 1.7 times for TPU10, TPU20 and TPU30, respectively. It suggests that highly porous samples, TPU20 and TPU30, exhibited less sensitive dependence on strain, in comparison with TPU10. This means the high porosity could significantly reduce the interference of stretching operations on the conductivity and benefit the stability of the electrical conductive performance of materials in the application of flexible electronics.

The relaxation behavior of each sample at different strains is shown in Fig. 6c. The sample was stretched to a desired strain (*e.g.* 5% and 10%) at a speed of 50 mm/min and then waited for 1 min before further stretching. It is apparent that all the samples had a remarkable creep and the relaxation amplitude increased with increasing strain. Taking TPU30 for an example, the relative resistance relaxed by 0.11 at 5% strain and 0.35 at 30% strain. This overshoot behavior was commonly observed in conductive elastomer composites^43,44^. This was caused by the relaxation of conductive networks in conductive elastomer nanocomposites, which was expected to be controlled by the relaxation behavior of elastomer matrix. Therefore, to determine the relaxation time, the experimental relaxation curves of the CNF/TPU nanocomposites as a function of time (*t*) at different strains were fitted with the stretched exponential Kohlrausch’s Eq. (1)^45^.

1 $$Normalized\,resistance\,R/{R}_{0}\,={R}_{\infty }+{R}_{1}{e}^{-{(\frac{t}{\tau })}^{\beta }}$$

**Figure 7:**
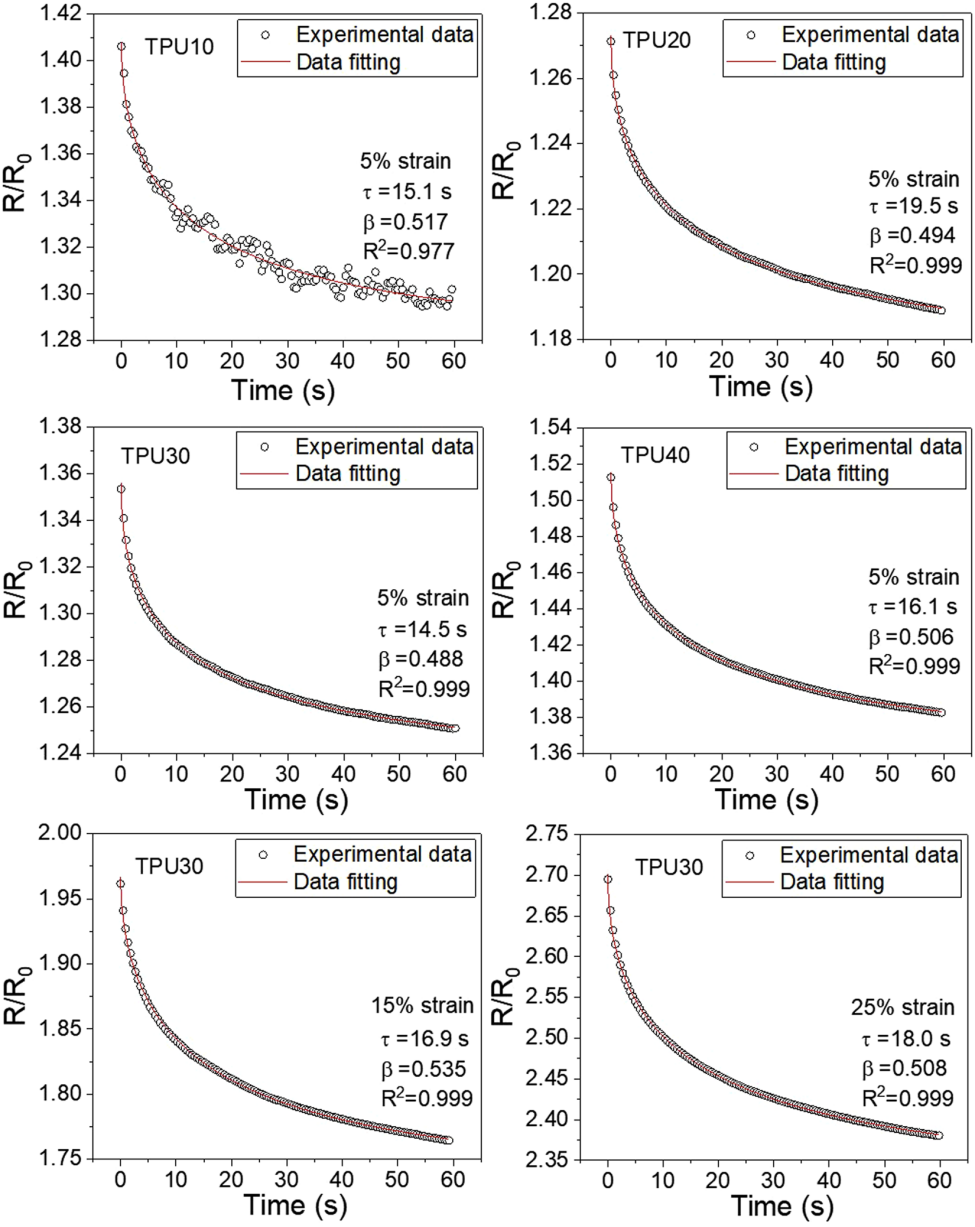
Time dependence on the normalized resistance for CNF/TPU nanocomposites at different strains.

Here, *R*_*∞*_, *R*_1_, *τ*, and *β* refer to the fitting constants. *τ* is the relaxation time and *β* is the stretching parameter (0 < *β* ≤ 1). The experimental data were well fitted with the theoretical values and the obtained relaxation times are given in Fig. 7. The relaxation time reflects the responding rate of the sample to reach the stable resistance. It can be seen the relaxation time at 5% strain for each sample was in the range of 14–20 s. The differences in relaxation time might be related to the different porous structures in nanocomposites and the different CNF loadings. The relaxation time increased with increasing strain (For TPU30, the relaxation time was 14.5 s at 5% strain, 16.9 s at 15% strain, and 18.0 s at 25% strain), because of the non-equilibrium rate dependent response of TPU. That is consistent with previous studies on stress relaxation behavior of TPU^4,46^.

**Figure 8:**
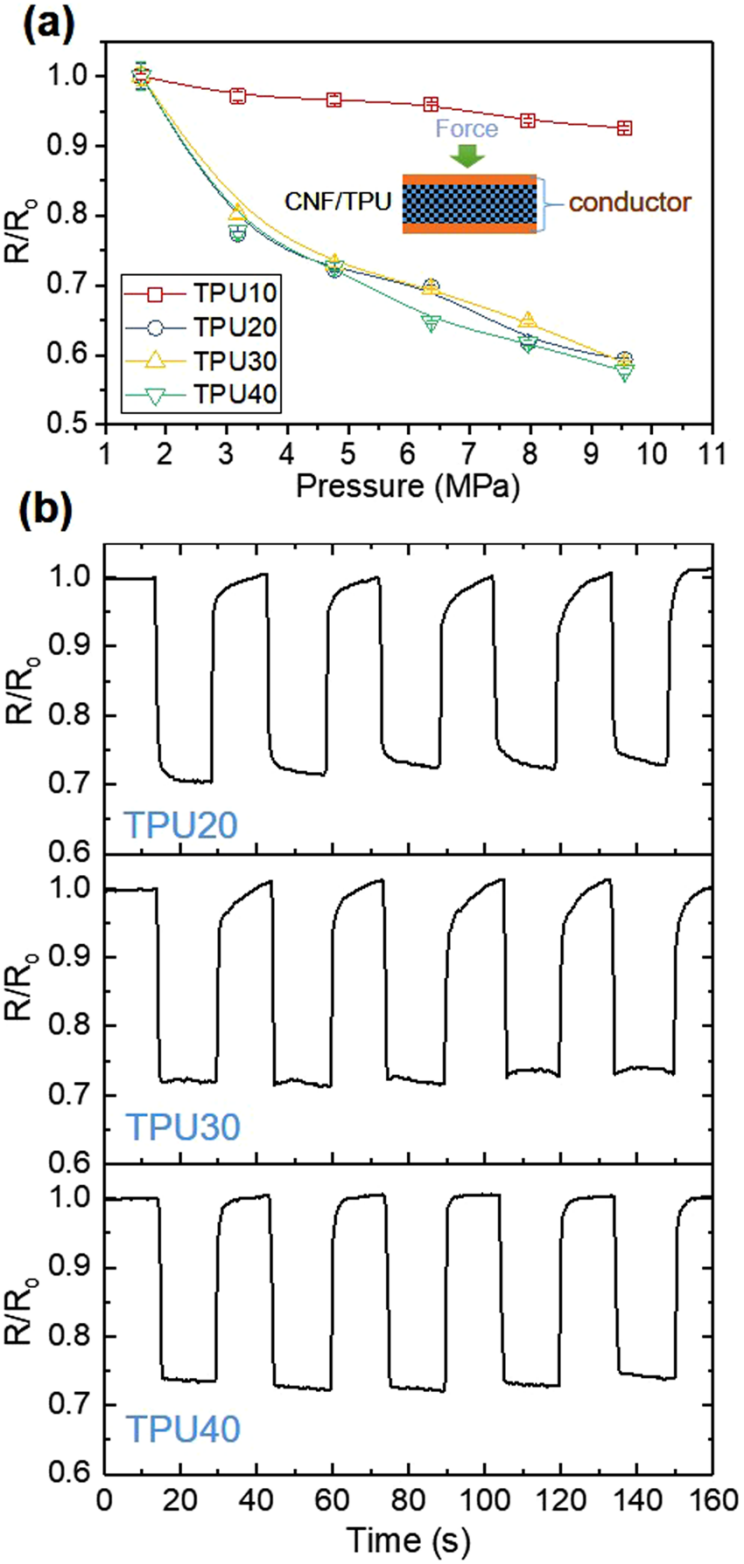
Piezoresistive properties of porous CNF/TPU nanocomposites: the normalized resistance versus pressure (**a**) and the normalized resistance versus time for the as-established device in response to pulse pressure application of approximately 4.8 MPa. The inset of (**a**) is the schematic setup for the pressure-responsive test.

Conductive elastomer composites usually demonstrate the piezoresistive effect, owing to the raised electrical conductivity caused by the compression-induced reduction in the thickness of insulating polymer layer^47^. The piezoresistive effect in CNF/TPU nanocomposites was investigated, as shown in Fig. 8a. The device setup with a sandwich configuration was shown in the inset of Fig. 8a. As anticipated, the resistance of TPU10 decreased with increasing pressure in the range from 1.6 MPa pressure up to 9.5 MPa. The resistance was reduced by ~10% at 9.5 MPa. Highly porous CNF/TPU nanocomposites (*i.e*. TPU20, TPU30 and TPU40) demonstrated stronger piezoresistive effects in comparison to TPU10. For instance, the resistance reductions for TPU20, TPU30 and TPU40 were approximately 40% at 9.5 MPa. To demonstrate repeatability of the piezoresistive behavior, dynamic pressure sensitivity was monitored while measuring the resistance variations with the repeated application of ~4.8 MPa for five cycles (Fig. 8b). Under a compression of ~4.8 MPa, the measured resistance becomes noticeably lower; once the force was released, the resistance gradually increased to the initial value. The as-established device exhibited a significant change in the resistance and good reproducible dynamic responses.

## Conclusions

Porous conductive TPU nanocomposites were successfully prepared by a conventional solution-casting method. As a rigid conductive filler with high aspect ratio, CNFs were utilized to cement the porous structure in the water vapor-induced TPU organogel. The CNF loading played an important role in the formation of final porous structures in TPU nanocomposites. With a CNF loading lower than 10 wt.%, the CNT-reinforced TPU-phase framework was not strong enough to withstand the shrinkage caused by DMF evaporation. With increasing CNF loading higher than 20 wt.%, the porous structures in TPU nanocomposites became well-developed. The porosity significantly increased with increasing CNF loading to 20 wt.% and reached a plateau at approximately 80%. The electrical conductivity of TPU nanocomposites increased with the CNF loading. The highest conductivity was achieved as 2.98 × 10^−2^ S/cm with 40 wt.% CNFs. The conductivity for all nanocomposite samples exhibited relaxation behavior due to the viscoelasticity of TPU elastomer. The mechanical properties of highly porous samples were different from the solid samples. TPU30 presented the best specific stiffness. All nanocomposite samples showed piezoresistive behavior. Highly porous CNF/TPU nanocomposites demonstrated stronger piezoresistive effect in comparison to TPU10, exhibiting good reproducible dynamic responses in dynamic tests. This study provides a novel and facile approach to produce lightweight conductive polymer films which have potential in pressure sensors, electromagnetic interference shielding, and filtration membranes.

## Methods

### Materials

Thermoplastic polyurethane elastomer (IROGRAN PS 455-203) was obtained from Huntsman, which has a Shore A hardness of 78 and density of 1.19 g/cm^3^. Carbon nanofibers (outer diameter: 100 nm and length: 20–200 μm) and N,N-dimethylformamide (99.8%) were purchased from Sigma-Aldrich. All materials were used as received.

### Preparation of porous CNF/TPU nanocomposites

CNF/TPU nanocomposites with various concentrations were prepared by solution blending followed by solution casting. Firstly, CNFs (0.3 g) were dispersed into 15 mL DMF by sonication in an ultrasonic bath (Fisherbrand 15051) for 30 min. Then, a desired amount of TPU was weighed and dissolved in the suspension under magnetically stirring for 5 hours. The resultant mixture was poured into a polytetrafluoroethylene (PTFE) dish and was kept in a fume cupboard for 2 h to allow the formation of TPU DMF organogel at a relative humidity of ~80% and for another 22 h to allow the evaporation of DMF. The residual solvent was removed in vacuum at 40 °C for 24 h. The residual solvent was removed in vacuum at 40 °C for 24 h. Samples with 0%, 10%, 20%, 30% and 40% mass percentages of CNFs were obtained by this method and designated as TPU, TPU10, TPU20, TPU30 and TPU40, respectively.

### Characterization

Attenuated total reflectance-Fourier transform infrared (ATR-FTIR) spectroscopy was carried out on a Frontier Optica spectrophotometer (PerkinElmer). The wavenumber region was between 4000 to 600 cm^−1^ with a resolution of 1 cm^−1^. The cross-section and top surface of TPU and CNF/TPU nanocomposites were investigated by scanning electron microscopy (SEM) on Inspect F (FEI) using a 10 kV acceleration voltage. For cross-sectional imaging, the samples were fractured in liquid nitrogen and coated with gold prior to SEM observations. Tensile tests were carried out at room temperature (25 °C) on a Hounsfield universal testing machine (Tinius Olsen Ltd.), where a 10 N load cell and a 100 mm/min testing speed were employed. For each sample, five specimens were tested. The electrical properties of the sample were monitored using 4-point probes method via a benchtop Agilent 34401 A multimeter (Keysight Technologies Inc.). For the conductivity test, a disk-like sample (*Φ*16 mm) was used and contacted to the circuit via two plate aluminium electrodes. For the piezoresistivity measurement, the resistance of the sample was recorded under various external pressures applied by the Hounsfield universal testing machine. The porosity (void fraction) of samples was calculated from the true density measured on AccuPyc-II-1340 pycnometer (Micromeritics Instrument Corp.) and the bulk density.

### Data Availability Statement

The datasets generated during and/or analysed during the current study are available from the corresponding author on reasonable request and shared in Zenodo repository.

## Electronic supplementary material

Supplementary Information
