# Supplementary material for: Facile Fabrication of Porous Conductive Thermoplastic Polyurethane Nanocomposite Films via Solution Casting
Source: Sci Rep. 2017 Dec 12;7:17470. doi: 10.1038/s41598-017-17647-w (PMC5727098; doi:10.1038/s41598-017-17647-w)
Supplement: Supplementary file 1 — Supplementary Information [file 41598_2017_17647_MOESM1_ESM.pdf]

Supplementary Information for:

Facile Fabrication of Porous Conductive Thermoplastic Polyurethane Nanocomposite Films *via* Solution Casting

Tongfei Wu[1] and Biqiong Chen[1,2]*

[1]Department of Materials Science and Engineering, University of Sheffield, Mappin Street, Sheffield S1 3JD, United Kingdom

[2]School of Mechanical and Aerospace Engineering, Queen's University Belfast, Stranmillis Road, Belfast BT9 5AH, United Kingdom

*b.chen@qub.ac.uk

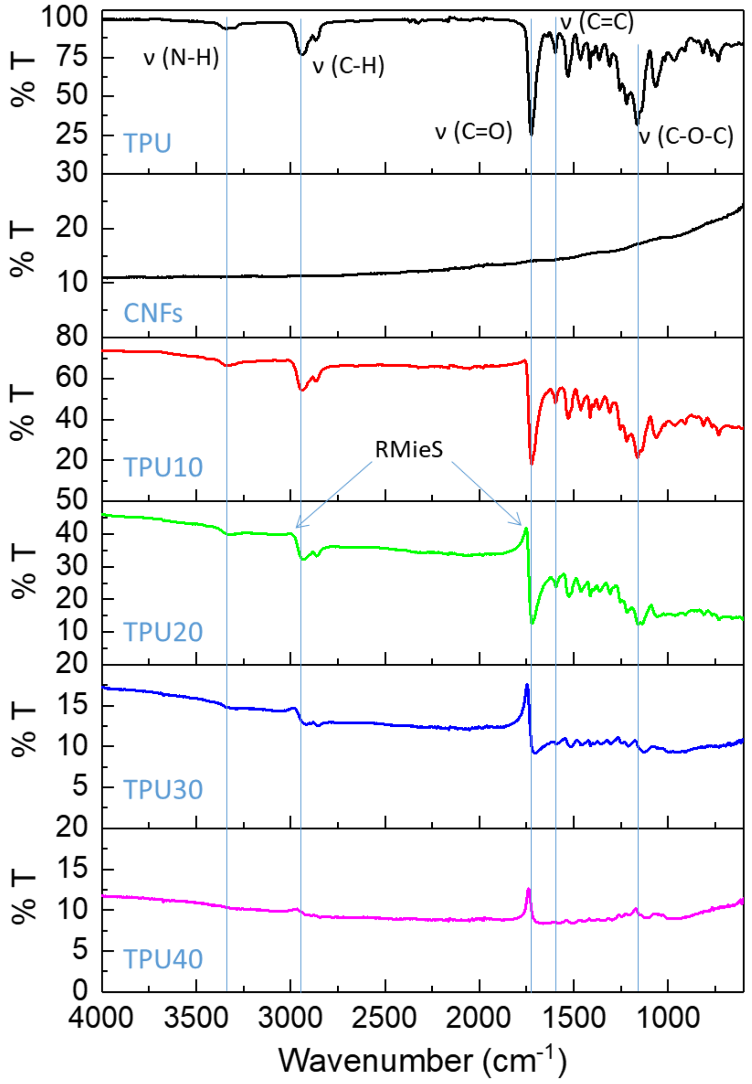

Figure S1. FTIR spectra of TPU, CNFs and CNF/TPU nanocomposites, where *v* refers to the stretching mode.

Table S1. Assignments of FTIR bands of TPU.

Wavenumber (cm^{-1})	Assignment[1, 2]
3330, 3340	N-H stretching in carbamate
2970, 2950	C-H stretching
1730	C=O stretching in carbamate
1596	C=C stretching in benzene ring of the rigid isocyanate segment
1533	amide II
1464	C-H asymmetric bending in CH_2
1413	CH_2 scissoring
1360	CH_3 wagging
1309	aromatic C-N vibration
1254	amide III
1221	symmetric C-O-C stretching in ether group
1160	C-O-C stretching in polyether diol

References

1. N. Luo, D. N. Wang and S. K. Ying, *Macromolecules*, 1997, **30**, 4405-4409.
2. S. Todros, C. Venturato, A. N. Natali, G. Pace and V. Di Noto, *J. Polym. Sci., Part B: Polym. Phys.*, 2014, **52**, 1337-1346.
